# Comparable medial meniscus extrusion in posterior root tears and radial tears with complex tears

**DOI:** 10.1002/jeo2.70544

**Published:** 2025-11-14

**Authors:** Tomoya Iseki, Shintaro Onishi, Ryo Kanto, Yoshitaka Nakao, Hiroki Miya, Akira Kawai, Takuya Iseki, Shinichi Yoshiya, Toshiya Tachibana, Hiroshi Nakayama

**Affiliations:** ^1^ Department of Orthopedic Surgery Hyogo Medical University Nishinomiya Japan; ^2^ Nishinomiya Kaisei Hospital Nishinomiya Japan

**Keywords:** horizontal tear, medial meniscal posterior root tear, meniscal extrusion, radial tear

## Abstract

**Purpose:**

To elucidate the relationship between medial meniscus extrusion (MME) and meniscal tear type in osteoarthritic knees. It was hypothesised that the type of meniscal tears in osteoarthritic knees would be relevant factors in triggering MME.

**Methods:**

Patients with medial compartmental knee osteoarthritis were retrospectively reviewed. MME was defined as the distance from the extruding edge of the meniscus to a line drawn perpendicularly from the medial edge of the tibial plateau. Meniscal tears were classified into four types on magnetic resonance imaging: horizontal tear, medial meniscus posterior root tear (MMPRT), small radial tear and radial tear combined with a complex tear. A multivariate logistic regression analysis was performed to identify risk factors associated with pathological MME.

**Results:**

A total of 153 patients with 58 ± 8 years were included. The averages amount of MME values were 3.4 ± 1.9 mm for horizontal tears (*n* = 42), 5.0 ± 1.3 mm for MMPRTs (*n* = 25), 4.2 ± 2.0 mm for small radial tears (*n* = 20) and 5.0 ± 2.2 mm for large/complex radial tears (*n* = 66). The amount of MME for MMPRTs and radial tear combined with complex tear was significantly greater than that of horizontal tears (*p* = 0.001, *p* < 0.0001, respectively). In multivariable analysis, age, MMPRT and radial tear combined with complex tear were identified as significant risk factors associated with MME greater than 3 mm (*p* = 0.006, 0.003 and 0.013; odds ratio = 1.09, 10.70 and 3.03, respectively).

**Conclusions:**

The presence of a radial tear combined with a complex tear was significantly correlated with MME, similar to MMPRT. This finding indicates that radial tears, which extend in multiple directions, may increase the risk of MME. Therefore, radial tears warrant careful evaluation, as they may carry a risk of MME comparable to that of MMPRTs.

**Level of Evidence:**

Ⅳ, retrospective case study

**Abbreviations**
BMI: body mass index.
HKA: hip‐knee‐ankle angle
JLCA: joint line convergence angle
JLO: joint line obliquity
KL: Kellgren and Lawrence
mLDFA: mechanical lateral distal femoral angle
MME: medial meniscus extrusion
MMPRT: medial meniscus posterior root tear
mMPTA: mechanical medial proximal tibial angle
OA: osteoarthritis
%MA: percentage of mechanical axis

## INTRODUCTION

Medial meniscus extrusion (MME) has been reported to be a strong predictor for the development and progression of varus knee osteoarthritis (OA) [2, 4, 10, 15, 20]. Previous studies addressing this subject have reported that various factors are associated with MME, including meniscal tears, mechanical alignment, cartilage damage and joint space narrowing [7, 9, 10, 13]. Of these factors, the relationship between medial meniscal tears and MME has been investigated and reported in the literature [1, 4, 6, 16]. Although it remains controversial whether MME precedes OA progression or whether OA itself leads to MME, their coexistence has been associated with increased functional decline and a higher risk of varus knee OA. Therefore, it is clinically important to understand the correlation between medial meniscal tears and MME, as surgical treatment to repair the meniscal tear and reduce the extruded meniscus can be the key to preventing knee OA.

There are several types of meniscal tears in knees with degenerative changes. Among them, medial meniscal posterior root tear (MMPRT) is a significant cause of MME [5]. Additionally, large radial tears have a poor prognosis. Complete radial tears extending to the periphery significantly reduce meniscal function, leading to cartilage damage. In addition to root and radial tears, there are several other different modes of degenerative tears, including horizontal and complex tears. Although several studies have reported comparisons of MME between various tear types [1, 6, 16], the relevance of radial tear in the development of MME has not yet been fully clarified. As a result, there is no consensus on the optimal management strategy for degenerative medial meniscal tears.

Given that the aetiology of MME is multifactorial including not only meniscal tears but also contributions from osteophyte formation, the joint capsule, and surrounding ligaments, elucidating the relationship between meniscal tears and MME remains of significant clinical relevance. The purpose of this study was to elucidate the relationship between MME and meniscal tear type in osteoarthritic knees. It was hypothesised that the type of meniscal tears in OA knees would be relevant factors in triggering MME.

## MATERIALS AND METHODS

### Study population

The design of this retrospective study was approved by the Institutional Review Board (No. 2217) and written informed consent was obtained prior to date collection from all patients. Patients were eligible for inclusion if they were aged 50 years or older, diagnosed with medial compartmental OA of the knee, and presented to our outpatient clinic between April 2021 and March 2022 with persistent medial knee pain lasting more than three months despite undergoing conservative treatment. All patients were required to have both preoperative plain radiographs and magnetic resonance imaging (MRI) data available for analysis. All included patients underwent MRI either as part of the routine clinical evaluation at our institution or prior to referral. Whether the patient had a history of previous knee trauma has not clearly documented. Exclusion criteria were (1) absence of MRI data; (2) absence of clear radiographic OA (KL grade ≤1); (3) younger than 50 years of age; (4) severe joint deformity corresponding to Ahlbäck classification grade >3, even among patients with KL grade 4 OA, due to difficulty in identifying the medial tibial edge for accurate MME assessment on MRI; and (5) presence of a clearly documented history of significant knee trauma, as this study aimed to evaluate atraumatic degenerative cases.

### MRI and radiological evaluation

**Figure 1:**
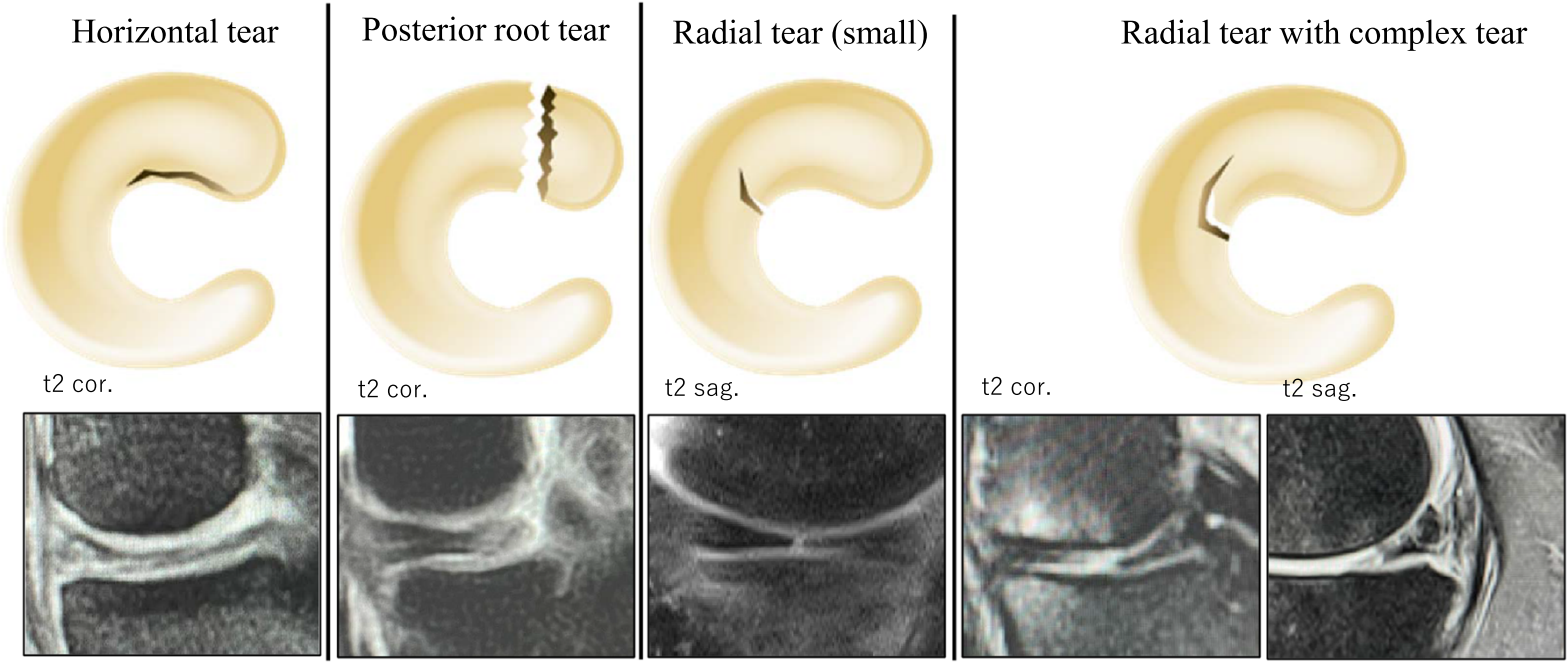
Magnetic resonance imaging assessment of meniscal tear type; magnetic resonance imaging. Cor; clonal. Sag, sagittal.

Meniscal tears were classified into four types: horizontal tear, MMPRT, small radial tear and radial tear combined with complex tear as detected on MRI. MRI images were classified based on specific signal patterns to identify each of the different types of meniscal tears. MMPRT was diagnosed when a high signal intensity line or discontinuity of the meniscal image was observed at the posterior meniscal attachment on a coronal image. A horizontal tear was indicated by a high signal intensity line extending horizontally to the articular surface. A small radial tear was identified by a high signal intensity line transecting the meniscal body on a consecutive two slice image in any of the sagittal, coronal, or axial planes. Tears with radial orientation accompanied by longitudinal, flap or oblique components were categorised as radial + complex tears. This is a pragmatic classification used to distinguish them from simple radial tears. Finally, a radial tear was classified as complex if high signal lines were detected on both the sagittal and coronal planes of the MRI (Figure 1). This categorisation was pragmatically adopted by the authors to distinguish complex radial tears with biomechanical significance from smaller, isolated tears.

**Figure 2:**
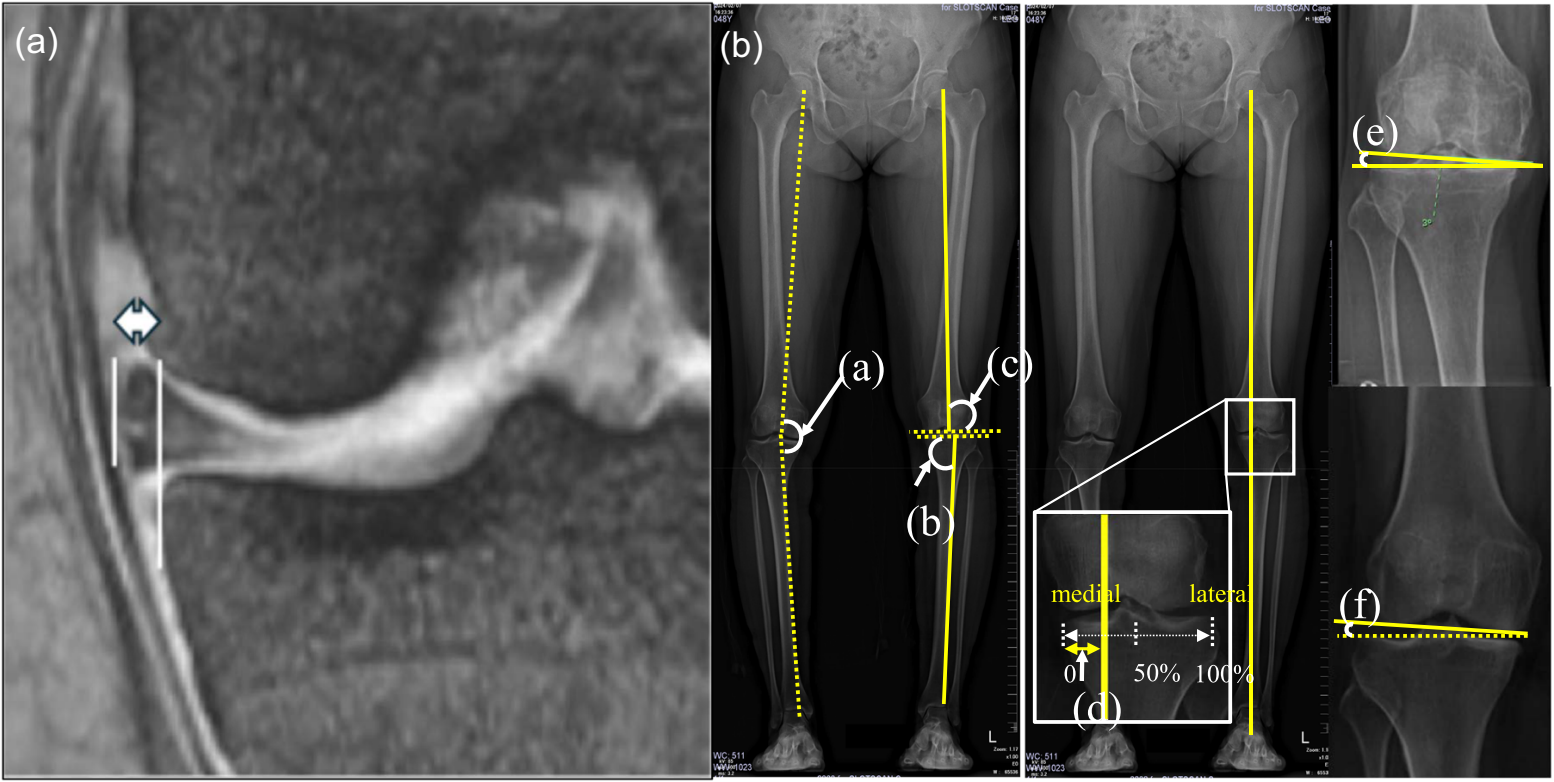
(A) Measurement of MME on the coronal MRI image. The measurement of meniscal extrusion was conducted by determining the distance from the edge of the extruded meniscus to a parallel line drawn perpendicularly from the medial tibial margin, excluding the bone spur. (B) Measurement of radiological parameters on a long‐leg radiograph. (a) Hip–knee–ankle angle (HKA). (b) Mechanical medial proximal tibial angle (mMPTA). (c) Mechanical lateral distal femoral angle (mLDFA). (d) Percentage of mechanical axis (%MA). (e) Joint line convergence angle (JLCA). (f) Joint line obliquity angle (JLO). MME, medial meniscus extrusion; MRI, magnetic resonance imaging.

The amount of MME was measured by identifying the image slice showing maximum meniscal extrusion on a coronal proton‐density‐weighted image [6, 7]. The distance from the extruding edge of the meniscus to a parallel line drawn perpendicularly from the medial tibial edge, excluding the bone spur, was then measuredand defined as the amount of meniscal extrusion (Figure 2a).

For the radiological assessment of bone/joint morphology and limb alignment, Hip–knee–ankle angle (HKA), percentage of mechanical axis (%MA), mechanical lateral distal femoral angle (mLDFA), mechanical medial proximal tibial angle (mMPTA), joint line obliquity (JLO) and joint line convergence angle (JLCA) were measured using a whole‐leg radiograph. The %MA was defined as the horizontal distance from the weight‐bearing Line (WBL) to the medial edge of the tibial plateau, expressed as a percentage of the tibial plateau width (with 0% being the medial edge and 100% being the lateral edge; Figure 2b).

### Statistical analysis

Normality of the data distribution was evaluated using the Shapiro‐Wilk test; differences in the amount of MME among the four types of meniscal tears and KL classification grades were statistically assessed by analysis of variance. In addition, correlations between patient characteristics and radiographic parameters, which included HKA, mLDFA and mMPTA along with the degree of MME, were evaluated using Pearson's correlation coefficient. A multivariate logistic regression analysis of risk factors for the development of the pathological MME ( > 3 mm) was also performed, with the 3‐mm threshold defined according to the criteria reported by Makiev et al [17].

Meniscal tear types, MME on MRI, and radiographic evaluations including the Ahlbäck classification and the Kellgren–Lawrence scale were taken twice, 2 weeks apart, and conducted by two evaluators (T.I. and H.M.), both of whom have over 5 years of clinical experience in musculoskeletal imaging and knee joint disorders. Intra‐ and interobserver reliability was assessed by the intraclass correlation coefficients (ICCs) using JMP Pro (ver. 13.0.0; SAS, NC, USA). The ICC values calculated for the interobserver reliabilities of the MME measurements taken on MRI and the radiological parameters representing %MA were 0.92 (%95 CI [0.89–0.94])/0.91 (%95 CI [0.87–0.94]) and 0.92 (%95 CI [0.89–0.94])/0.92(%95 CI [0.89–0.94]), indicating excellent interobserver reliability, while the intraobserver reliabilities were 0.82 (%95 CI [0.78–0.86]) and 0.92 (%95 CI [0.89–0.94]), indicating good to excellent intraobserver reliability. The standard error of measurement (SEM) for MME was 0.89 mm (intraobserver) and 0.59 mm (interobserver), while the SEM for %MA was 3.39%. In order to evaluate the statistical power of this study, a post hoc power analysis was performed using G*Power (version 3.1.9.2; Faul et al., Heinrich Heine University, Düsseldorf, Germany). Based on the observed sample sizes for the large/complex radial tear and horizontal tear groups, the analysis demonstrated adequate power (1 – β = 0.815) at a significance level of α = 0.05.

## RESULTS

**Figure 3:**
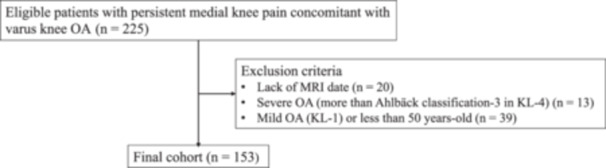
Flowchart showing selection of study cohort. KL, Kellgren–Lawrence grade; MRI, magnetic resonance imaging; OA, osteoarthritis.

Of the initial 225 patients assessed, 72 who met the exclusion criteria were removed, resulting in a final cohort of 153 patients included in the study based on the predefined inclusion and exclusion criteria. A flowchart of the study cohort is shown in Figure 3. Demographic characteristics of the patients are presented in Table 1.

**Table 1:** Demographic profile of the study population.

Variable	Value
Total patients, *n*	153
Age (y)	58.0 ± 7.5
Sex, male/female, *n*	76/77
Laterality, right/left, *n*	73/80
Amount of MME (mm)	4.4 ± 2.1
MME < 3 mm/MME > 3 mm	35/118
Horizontal/posterior root/radial (small)/radial (large/complex), *n*	42/25/20/66
KL classification 2/3/4 (excluding Ahlbäck classification > grade 3), *n*	54/70/29
%MA (%)	26.6 ± 12.0
HKAA (°)	Varus 5.3 ± 3.4
MPTA (°)	84.9 ± 2.5
mLDFA (°)	87.5 ± 1.8
JLO (°)	1.4 ± 3.0
JLCA (°)	2.9 ± 2.0

Abbreviations: BMI, body mass Index; HKAA, hip–knee–ankle angle; JLCA, joint line convergence angle; JLO, joint line obliquity; KL, Kellgren–Lawrence; mLDFA, mechanical lateral distal femoral angle; MME, medial meniscus extrusion; MPTA, medial proximal tibial angle; %MA, percentage of mechanical axis.

**Figure 4:**
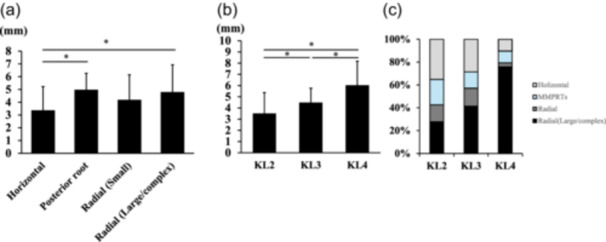
(a) The amount of MME associated with different types of meniscal tears. (b) The amount of MME related to KL classification. *Statistically significant difference between groups (*p* < 0.05). KL, Kellgren–Lawrence; MME, medial meniscus extrusion.

The average amount of MME was 4.4 ± 2.0 mm, and ΔMME greater than 3 mm was detected in 116 of the 153 knees (75.8%) included in this study. With regard to the correlation between meniscal tear type and ΔMME, the average amount of MME values were 3.4 ± 1.9 mm for horizontal tears, 5.0 ± 1.3 mm for MMPRTs, 4.2 ± 2.0 mm for small radial tears, and 5.0 ± 2.2 for large/complex radial tears. With respect to radial tear, no statistically significant difference was observed between small radial tears and large/complex radial tears. The ΔMME for MMPRTs and large/complex radial tears was significantly greater than that of horizontal tears (*p* = 0.001, *p* < .0001; Figure 4a).

As for KL classification, the amount of MME averaged 3.5 ± 1.5 mm for KL‐2 knees, 4.5 ± 2.0 mm for KL‐3 knees, and 6.0 ± 2.3 mm for KL‐4 knees (Table 1 **)**. When compared between cohorts of different KL grades, the amount of MME in KL‐3 knee1s was significantly greater than in KL‐2 knees, and ΔMME in KL‐4 knees was significantly greater than in KL‐3 knees (*p* = 0.013 and *p* < 0.001, respectively; Figure 4b).

The ratio of meniscal tear types in KL classification is illustrated in Figure 4c. For KL‐2, the distribution of tear types was as follows: horizontal tears constituted 35.2% (19 knees), MMPRTs accounted for 22.2% (12 knees), small radial tears represented 14.8% (8 knees) and large/complex radial tears comprised 27.8% (15 knees). For KL‐3, horizontal tears were 28.6% (20 knees), MMPRTs were 14.3% (10 knees), small radial tears were 15.7% (11 knees) and large/complex radial tears were 41.4% (29 knees). For the ratio of KL‐4, horizontal tears and MMPRTs were each 10.3% (3 knees), while small radial tears accounted for 3.5% (1 knee) and large/complex radial tears made up 75.9% (22 knees). A statistically significant association between meniscal tear type and KL classification was identified for complex tears (*p* < 0.001). In contrast, no statistically significant differences in KL grade distribution were observed for the other tear types, small radial tears, horizontal tears and MMPRTs.

Stratification based on MME threshold of 3 mm identified a predominance of large or complex radial tears in the MME ≥ 3 mm group (*n* = 116), with 53 cases (45.7%). Horizontal tears and medial meniscus posterior root tears (MMPRT) were each observed in 24 cases (20.7%), followed by small radial tears in 15 cases (12.9%). In the group with MME < 3 mm (*n* = 37), horizontal tears were the most common, observed in 18 cases (48.6%), followed by large/complex radial tears with 13 cases (35.1%), small radial tears with 5 cases (13.5%) and MMPRT with 1 case (2.7%).

Correlation coefficients of ΔMME with patient characteristics and radiological parameters are presented in Table 2. Significant correlations with the amount of MME were observed for age and varus deformity of the lower leg (%MA, HKAA and JLCA). Subsequent multivariable analysis for risk factors for MME did not identify varus deformity of the lower leg (HKAA) and KL classification as significant independent risk factors. On the other hand, age, MMPRT and large/complex radial tears were identified as significant risk factors associated with MME greater than 3 mm (Table 3).

**Table 2:** Correlation of the amount of MME with patient characteristics and radiological parameters.

	Amount of MME (mm)	
Variables	Correlation coeffect	*p* Value
Age	0.31	0.0001
BMI	0.001	0.91
%MA (%)	−0.28	0.0006
HKAA (°)	0.32	<0.0001
MPTA (°)	−0.13	0.16
mLDFA (°)	0.16	0.06
JLO (°)	−0.08	0.31
JLCA (°)	0.19	0.02

Abbreviations: %MA, percentage of mechanical axis; BMI, body mass index; HKAA, hip knee ankle angle; JLCA, joint line convergence angle; JLO, joint line obliquity; mLDFA, mechanical lateral distal femoral angle; MME, medial meniscus extrusion; MPTA, medial proximal tibial angle.

**Table 3:** Multivariate logistic regression analysis of risk factors for MME.

		MME (>3 mm)	
Variables	Odds ratio	95% Confidence interval	*p* Value
Age	1.087	1.022–1.159	0.006
HKAA (°)	1.099	0.952–1.271	0.202
KL classification	1.235	0.499–3.175	0.652
Posterior root tear	10.7	1.984–201.3	0.003
Radial tear (large/complex)	3.03	1.226–7.696	0.013

Abbreviations: HKAA, hip knee ankle angle; KL, Kellgren–Lawrence; MME, medial meniscus extrusion.

## DISCUSSION

The results of present study indicated that a radial tear progressing to a complex tear configuration significantly correlated with MME, comparable to MMPRT.

Risk factors for MME have been examined in previous studies and several associated factors have been reported, including older age, high BMI, severe cartilage damage, varus deformity greater than 5°, and a longer time duration between injury and surgery [14, 18]. In addition, meniscal tears are also a known factor related to MME. Regarding the correlation between type of meniscal tear and MME, Crema et al. reported that 57.8% of knees with MME had concomitant meniscal tears, and the higher severity of the tear, the stronger the association with MME. Among the different tear types, MMPRT is known to be a strong cause of MME, and surgical repair of MMPRT is indicated for the purpose of preventing MME progression [11, 14]. The present study showed that the presence of a large/complex radial tear is also a risk for developing MME. In a biomechanical study comparing the effect of posterior root and radial tears on load distribution and transmission functions in the lateral meniscus, a root tear represented a higher contact pressure than a radial tear. In clinical studies comparing these two tear types, Costa et al. reported that the risk of MME was higher in MMPRT compared to radial tears, while Lee et al. showed that the incidence and extent of MME were similar in knees with MMPRT and radial tears.

As the incidence of traumatic radial tear is higher in the lateral meniscus due to injury during the cutting motion or combined injury with an anterior cruciate ligament injury, the relationship between radial tears and articular contact pressure has been widely investigated for the lateral meniscus [19]. Conversely, the biomechanical implications of a radial tear in the medial meniscus have not been thoroughly examined. Bedi A et al. conducted a human cadaveric experiment examining contact pressure transmitted to the medial tibial plateau under physiological load as a function of the percentage of the meniscus involved by the medial radial tear [3]. The study showed that a radial tear involving 30 and 60% of the width did not affect the status of the contact pressure. However, a tear involving 90% resulted in a shift in peak pressure location and an increase in pressure. Our study showed that radial tears, when aggravated to a complex tear by extending beyond the original tear site in another direction, were more frequently associated with MME than isolated radial tears.

There were several limitations included in the study. First, only MRI evaluation was used to assess meniscal tears in this study. As a result, this limited accurate identification of the direction and length of the tear. In the future, arthroscopic examination will be considered for more detailed evaluation of the tear type and extent. Second, some knees with advanced OA showing KL‐4 radiological changes were included in the analysis, which may have influenced the accuracy of the MME measurement. However, omitting the bone spur in the measurement and excluding knees with OA changes greater than Ahlbäck radiographic classification grade 3 may have contributed to retaining the intra‐ and interobserver reliabilities, as indicated by the relatively high ICC values. Third, despite prior reports suggesting a link between synovitis and MME, neither the presence nor the severity of synovitis was assessed in this study [8]. Fourth, MME was not normalised for tibial width, height, or BMI in this study. Although some previous studies have applied normalisation using these factors, many have reported direct measurements of MME in millimetres [12, 21]. Therefore, we adopted the latter approach in the present study.

## CONCLUSION

The present study showed that presence of a radial tear combine with complex tear was significantly correlated with MME, similarly to MMPRT. This finding indicates that radial tears, when extending in another direction, may elevate the risk of MME.

## AUTHOR CONTRIBUTIONS

All authors have contributed to the design, content and writing of the manuscript. All authors read and approved the final manuscript.

## CONFLICT OF INTEREST STATEMENT

The authors declare no conflict of interest.

## ETHICS STATEMENT

Ethical approval for this study was obtained from the Institutional Review Board in our institution (No. 2217). Informed consent was obtained from all individual participants included in the study.

## Data Availability

The data that support the findings of this study are available from the corresponding author upon reasonable request.
